# Supplementary material for: DeLUCS: Deep learning for unsupervised clustering of DNA sequences
Source: PLoS One. 2022 Jan 21;17(1):e0261531. doi: 10.1371/journal.pone.0261531 (PMC8782307; doi:10.1371/journal.pone.0261531)
Supplement: S1 Appendix — (PDF) [file pone.0261531.s001.pdf]

S1 Appendix

Instructions for reproduction of the tests using DeLUCS

First, go to https://github.com/millanp95/DeLUCS and clone the repository. Then run the series of commands for each test.

Test #1:

1. `python build_dp.py --data_path='../data/Vertebrata/Test Files'`
2. `python get_pairs.py --data_path='../data/Vertebrata/Test Files/train.p' --k=6 --modify='mutation' --output='../data/Vertebrata/Test Files/testing_data.p'`
3. `python EvaluateDeLUCS.py --data_dir='../data/Vertebrata/Test Files' --out_dir='../data/Vertebrata/Test Files'`

Comparison Models:

- `python EvaluateComparison.py --data_path='../data/Vertebrata/Test Files/testing_data.p'  --method='Supervised' --k=6`
- `python EvaluateComparison.py --data_path='../data/Vertebrata/Test Files/train.p'  --method='k-means++' --k=6`
- `python EvaluateComparison.py --data_path='../data/Vertebrata/Test Files/train.p'  --method='GMM' --k=6 --k=6`

Test #2:

1. `python build_dp.py --data_path='../data/Fish/Test Files/Actinopterygii'`
2. `python get_pairs.py --data_path='../data/Fish/Test Files/Actinopterygii/train.p' --k=6 --n_mimics=8 --modify='mutation' --output=../data/Fish/Test Files/Actinopterygii/testing_data.p'`
3. `python EvaluateDeLUCS.py --data_dir='../data/Fish/Test Files/Actinopterygii' --out_dir='../data/Fish/Test Files/Actinopterygii'`

Comparison Models:

- `python EvaluateComparison.py --data_path='../data/Fish/Test Files/Actinopterygii/testing_data.p'   --method='Supervised' --k =6`
- `python EvaluateComparison.py --data_path='.../data/Fish/Test Files/Actinopterygii/train.p'   --method='k-means++' --k=6`
- `python EvaluateComparison.py --data_path='../data/Fish/Test Files/Actinopterygii/train.p'   --method='GMM' --k=6`

Test #3:

```
1. python build_dp.py --data_path='../data/Fish/Test Files/Neopterygii'
2. python get_pairs.py --data_path='../data/Fish/Test
   Files/Neopterygii/train.p' --k=6 --modify='mutation'
   --output=../data/Fish/Test Files/Neopterygii/testing_data.p'
3. python EvaluateDeLUCS.py --data_dir='../data/Fish/Test Files/Neopterygii' --
   out_dir='../data/Fish/Test Files/Neopterygii'
```

Comparison Models:

```
• python EvaluateComparison.py --data_path='../data/Fish/Test
  Files/Neopterygii/testing_data.p'  --method='Supervised' --k =6
• python EvaluateComparison.py --data_path='../data/Fish/Test
  Files/Neopterygii/train.p'  --method='k-means++' --k=6
• python EvaluateComparison.py --data_path='../data/Fish/Test
  Files/Neopterygii/train.p'  --method='GMM' --k=6
```

Test #4:

```
1. python build_dp.py --data_path='../data/Fish/Test Files/Ostariophysi'
2. python get_pairs.py --data_path='../data/Fish/Test
   Files/Ostariophysi/train.p' --k=6 --n_mimics=8 --modify='mutation' --
   output=../data/Fish/Test Files/Ostariophysi/testing_data.p'
3. python EvaluateDeLUCS.py --data_dir='../data/Fish/Test Files/Ostariophysi'
   --out_dir='../data/Fish/Test Files/Ostariophysi'
```

Comparison Models:

```
• python EvaluateComparison.py --data_path='./data/Fish/Test
  Files/Ostariophysi/testing_data.p' --method='Supervised' --k =6
• python EvaluateComparison.py --data_path='../data/Fish/Test
  Files/Ostariophysi/train.p' --method='k-means++' --k=6
• python EvaluateComparison.py --data_path='../data/Fish/Test
  Files/Ostariophysi/train.p'  --method='GMM' --k=6
```

Test #5:

```
1. python build_dp.py --data_path='../data/Fish/Test Files/Cypriniformes'
2. python get_pairs.py --data_path='../data/Fish/Test
   Files/Cypriniformes/train.p' --k=6 --n_mimics=8 --modify='mutation' --
   output=../data/Fish/Test Files/Cypriniformes/testing_data.p'
3. python EvaluateDeLUCS.py --data_dir='../data/Fish/Test Files/Cypriniformes'
   --out_dir='../data/Fish/Test Files/Cypriniformes'
```

Comparison Models:

```
• python EvaluateComparison.py --data_path='../data/Fish/Test
  Files/Cypriniformes/testing_data.p'  --method='Supervised' --k =6
• python EvaluateComparison.py --data_path='../data/Fish/Test
  Files/Cypriniformes/train.p' --method='k-means++' --k=6
• python EvaluateComparison.py --data_path='../data/Fish/Test
  Files/Cypriniformes/train.p' --method='GMM' --k=6
```

Test #6:

1. python build_dp.py --data_path='../data/Fish/Test Files/Cyprinidae'
2. python get_pairs.py --data_path='../data/Fish/Test Files/Cyprinidae/train.p' --k=6 --n_mimics=8 --modify='mutation' --output=../data/Fish/Test Files/Cyprinidae/testing_data.p'
3. python EvaluateDeLUCS.py --data_dir='../data/Fish/Test Files/Cyprinidae' --out_dir='../data/Fish/Test Files/Cyprinidae'

Comparison Models:

- python EvaluateComparison.py --data_path='../data/Fish/Test Files/Cyprinidae/testing_data.p'   --method='Supervised' --k =6
- python EvaluateComparison.py --data_path='../data/Fish/Test Files/Cyprinidae/train.p' --method='k-means++' --k=6
- python EvaluateComparison.py --data_path='../data/Fish/Test Files/Cyprinidae/train.p' --method='GMM' --k=6

Test #7:

1. python build_dp.py --data_path='../data/Bacteria/Test_Files'
2. python get_pairs.py --data_path='../data/Bacteria/Test_Files/train.p' --k=6 --modify='mutation' --output='../data/Bacteria/Test_Files/testing_data.p'
3. python EvaluateDeLUCS.py --data_dir='../data/Bacteria/Test_Files' --out_dir='../data/Bacteria/Test Files'

Comparison Models:

- python EvaluateComparison.py --data_path='../data/Bacteria/Test_Files/testing_data.p' --method='Supervised' --k =6
- python EvaluateComparison.py --data_path='../data/Bacteria/Test_Files/train.p' --method='k-means++' --k=6
- python EvaluateComparison.py --data_path='../data/Bacteria/Test_Files/train.p' --method='GMM' --k=6

Test #8:

1. python build_dp.py --data_path='../data/Bacteria/Proteo_Test_Files'
2. python get_pairs.py --data_path='../data/Bacteria/Proteo_Test_Files/train.p' --k=6 --modify='mutation' --output='../data/Bacteria/Proteo_Test_Files/testing_data.p'
3. python EvaluateDeLUCS.py --data_dir='../data/Bacteria/Proteo_Test_Files' --out_dir='../data/ProteoBacteria/Test Files'

Comparison Models:

- python EvaluateComparison.py --data_path='../data/Bacteria/Proteo_Test_Files/testing_data.p' --method='Supervised' --k =6
- python EvaluateComparison.py --data_path='../data/Bacteria/Proteo_Test Files/train.p' --method='k-means++' --k=6
- python EvaluateComparison.py --data_path='../data/Bacteria/Proteo_Test_Files/train.p' --method='GMM' --k=6

Test #9:

1. python build_dp.py --data_path='../data/Influenza-A/Test Files'
2. python get_pairs.py --data_path='../data/Influenza-A/Test Files/train.p' --k=6 --modify='mutation' --output='../data/Influenza-A/Test Files/testing_data.p'
3. python EvaluateDeLUCS.py --data_dir='../data/Influenza-A/Test Files' --out_dir='../data/Influenza-A/Test Files'

   Comparison Models:

- python EvaluateComparison.py --data_path='../data/Influenza-A/Test Files/testing_data.p' --method='Supervised' --k =6
- python EvaluateComparison.py --data_path='../data/Influenza-A/Test Files/train.p' --method='k-means++' --k=6
- python EvaluateComparison.py --data_path=../data/Influenza-A/Test Files/train.p' --method='GMM' --k=6

Test #10:

1. python build_dp.py --data_path='../data/Dengue/Test Files'
2. python get_pairs.py --data_path='../data/Dengue/Test Files/train.p' --k=6 --modify='mutation' --output='../data/Dengue/Test Files/testing_data.p'
3. python EvaluateDeLUCS.py --data_dir='../data/Dengue/Test Files' --out_dir='../data/Dengue/Test Files'

   Comparison Models:

- python EvaluateComparison.py --data_path='../data/Dengue/Test Files/testing_data.p' --method='Supervised' --k =6
- python EvaluateComparison.py --data_path='../data/Dengue/Test Files/train.p' --method='k-means++' --k=6
- python EvaluateComparison.py --data_path='../data/Dengue/Test Files/train.p' --method='GMM' --k=6

Test #11:

1. python build_dp.py --data_path='../data/HBV/Test Files'
2. python get_pairs.py --data_path='../data/HBV/Test Files/train.p' --k=6 --modify='mutation' --output='../data/HBV/Test Files/testing_data.p'
3. python EvaluateDeLUCS.py --data_dir='../data/HBV/Test Files' --out_dir='../data/HBV/Test Files'

   Comparison Models:

1. python EvaluateComparison.py --data_path='../data/HBV/Test Files/testing_data.p' --method='Supervised' --k =6
2. python EvaluateComparison.py --data_path='../data/HBV/Test Files/train.p' --method='k-means++' --k=6
3. python EvaluateComparison.py --data_path='../data/HBV/Test Files/train.p' --method='GMM' --k=6

DeLUCS computational pipeline for running your own dataset:

1. Build the dataset:

```
python build_dp.py --data_path=<PATH_sequence_folder>
```

- Input: Folders with the sequences in FASTA format
- Output : file in the form (label,sequence,accession)

If the true label is unknown then place ALL the sequences in the same folder

2. Compute the mimics.

```
 python get_pairs.py --data_path=<PATH_dataset> --k=6 --modify='mutation' --
output=<PATH_output_file>
```

- Input: file in the form (label, sequence, accession)
- Output : file in the form of (pairs, x_test, y_test).

3. Train the model.

If the true labels are unknown for your dataset and you want to use DeLUCS as a clustering tool without the assessment by means of the Hungarian algorithm, run:

```
python TrainDeLUCS.py --data_dir=<PATH_of_computed_mimics> --out_dir=<OUTPURDIR> --
n_clusters=<expected_number_of_clusters>
```

- Input: file in the form of (pairs, x_test).
- Output : Cluster assignment for each sequence in x_test.

If the “ground truth” is known for your dataset and you want to train DeLUCS and then evaluate its performance by means of the Hungarian algorithm, run:

```
python EvaluateDeLUCS.py --data_dir=<PATH_computed_mimics> --out_dir=<OUTPURDIR>
```

- Input: file in the form of (pairs, x_test, y_test).
- Output : Image with the confusion matrix.
