# Supplementary material for: DeLUCS: Deep learning for unsupervised clustering of DNA sequences
Source: PLoS One. 2022 Jan 21;17(1):e0261531. doi: 10.1371/journal.pone.0261531 (PMC8782307; doi:10.1371/journal.pone.0261531)
Supplement: S2 Appendix — (PDF) [file pone.0261531.s002.pdf]

S2 Appendix

Query options for data download

Mitochondrial dataset (Nov 16, 2020) :
We used the software Geneious and the following keywords: *Mitochondrion*, *Mitochondria*, *Vertebrata* and *Complete Genome*.

Bacterial dataset (Jan 18, 2021):
We downloaded the file `bac120_taxonomy_r95.tsv` directly from GTDB https://data.gtdb.ecogenomic.org/releases/release95/95.0/ and filtered the families from the complete taxonomy.

Influenza A virus dataset (Oct 14, 2020):
The sequences were downloaded directly from:
https://www.ncbi.nlm.nih.gov/genomes/FLU/dataset/nph-select.cgi#mainform, using the following query options:

- sequence type: Nucleotide
- type: A
- subtypes: H1N1,H2N2, H5N1, H7N3, and H7N9
- segment: 6(NA)
- other options: default
- full length only
- collapse identical

-----------Dataset Statistics ------------
Total num of classes: 5
Total num of sequences: 13078
Min genome length: 52
Avg genome length: 1392.2
Max genome length: 1544
Data distribution:

H5N1	=>	3095
H2N2	=>	175
H1N1	=>	9189
H7N9	=>	293
H7N3	=>	326

Dengue virus dataset (Oct 14, 2020):

The sequences were downloaded directly from: https://www.ncbi.nlm.nih.gov/genomes/VirusVariation/dataset/nph-select.cgi?taxid=12637, using the following query options:

- sequence type: Nucleotide
- other options: default
- collapse identical
- full-length only

-----------Dataset Statistics ------------
Total num of classes: 5
Total num of samples: 5868
Min genome length: 10161
Avg genome length: 10582.002044989775
Max genome length: 11195
Data distribution:
Subtype-1 => 2446
Subtype-2 => 1891
Subtype-3 => 1121
Subtype-4 => 407
N/A => 3

Hepatitis B virus dataset (Oct 14, 2020):

The whole dataset was downloaded directly from:
https://hbvdb.lyon.inserm.fr/HBVdb/HBVdbDataset?seqtype=0.

-----------Dataset Statistics ------------
Total num of classes: 6
Total num of samples: 6493
Min genome length: 3182
Avg genome length: 3209.943015555213
Max genome length: 3254
Data distribution:
A => 880
B => 1765
C => 2194
D => 1090
E => 306
F => 258

Note: The curated datasets used to obtain the results in the paper can be found at:
https://github.com/millanp95/DeLUCS/tree/master/data
