# Supplementary material for: DeLUCS: Deep learning for unsupervised clustering of DNA sequences
Source: PLoS One. 2022 Jan 21;17(1):e0261531. doi: 10.1371/journal.pone.0261531 (PMC8782307; doi:10.1371/journal.pone.0261531)
Supplement: S3 Appendix — (PDF) [file pone.0261531.s003.pdf]

S3 Appendix

Confusion matrices

Figure 1 (Tests 1-6). Confusion matrices of the assignment that maximizes the accuracy at each taxonomic level of the mtDNA dataset. Predicted labels are numeric cluster assignments, omitted here for readability.

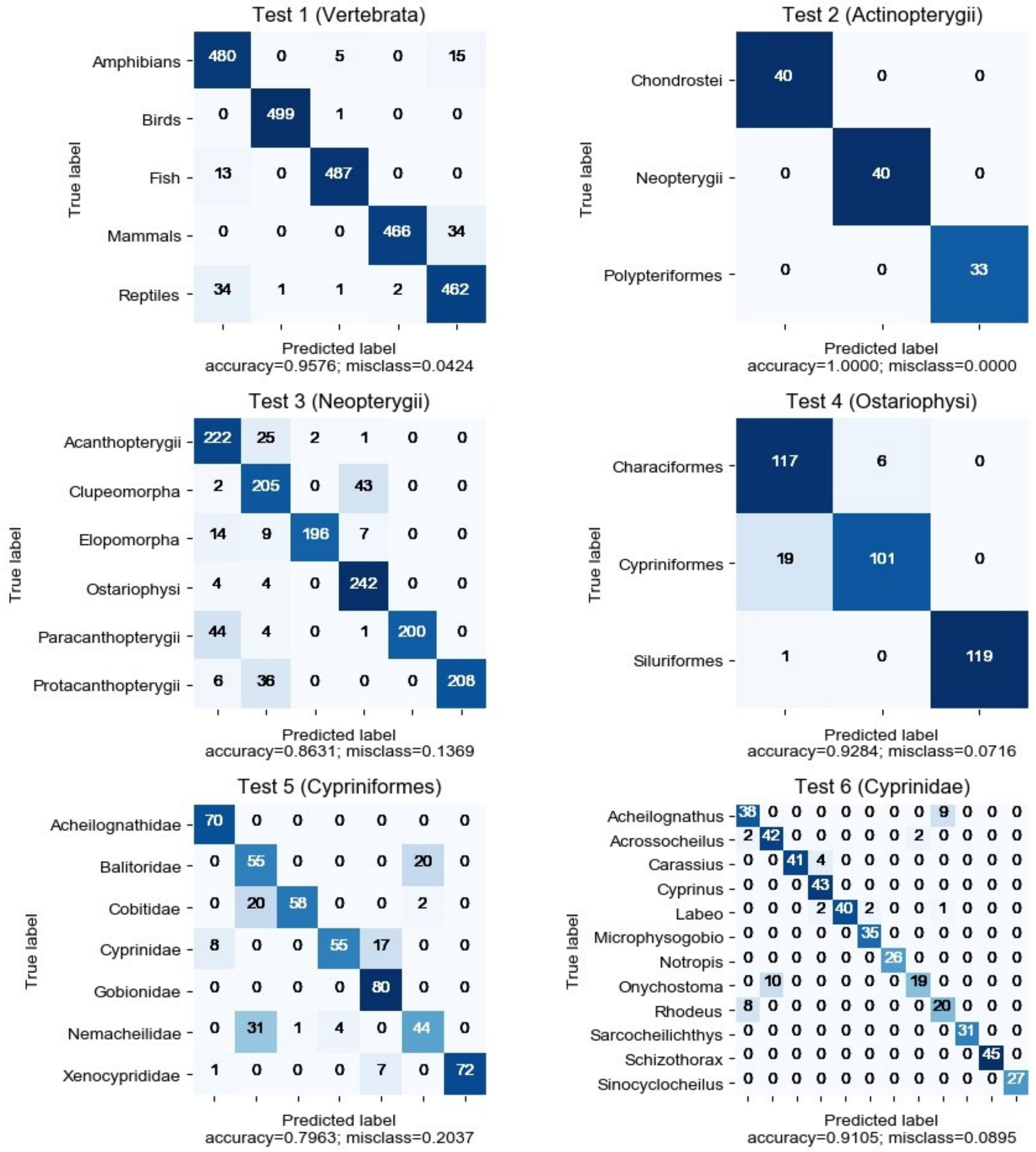

Figure 2 (Tests 7, 8). Confusion matrices of the assignment that maximizes the accuracy for both computational tests with the bacterial dataset at phylum level to families. (Top) All bacterial families

are considered. (Bottom) Only sequences in the phylum Proteobacteria are considered. Predicted labels are numeric cluster assignments, omitted here for readability.

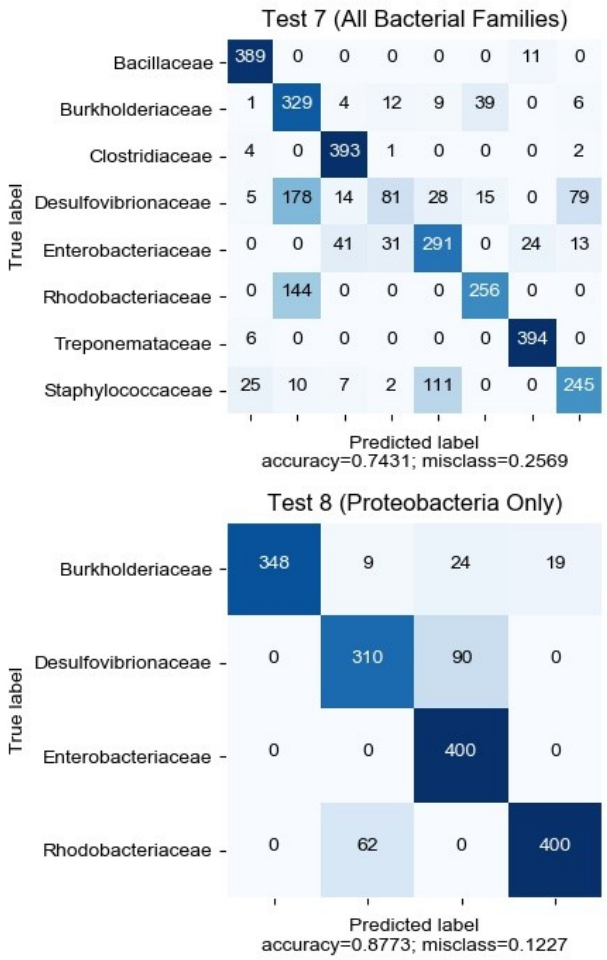

Figure (Tests 9-11). Confusion matrices of the assignment that maximizes the accuracy for the NA-encoding gene of the Influenza A virus, Dengue virus genomes, and HBV genomes. Predicted labels are numeric cluster assignments, omitted here for readability.

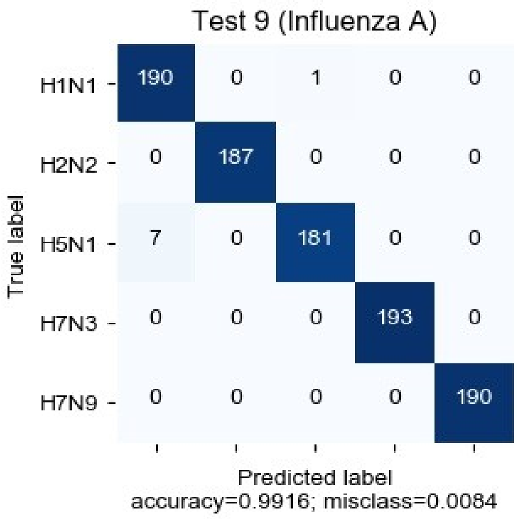
Test 9 (Influenza A)
True label
H1N1 190 0 1 0 0
H2N2 0 187 0 0 0
H5N1 7 0 181 0 0
H7N3 0 0 0 193 0
H7N9 0 0 0 0 190
Predicted label
accuracy=0.9916; misclass=0.0084

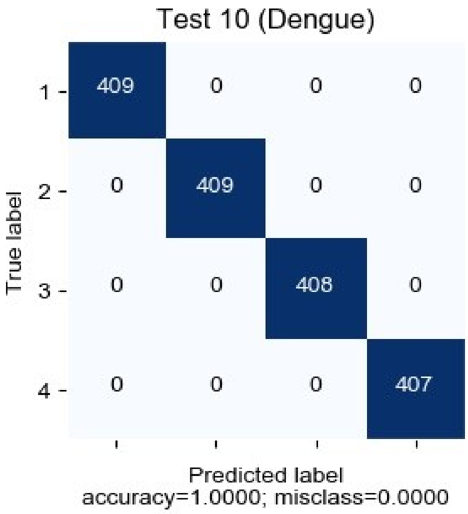
Test 10 (Dengue)
True label
1 409 0 0 0
2 0 409 0 0
3 0 0 408 0
4 0 0 0 407
Predicted label
accuracy=1.0000; misclass=0.0000

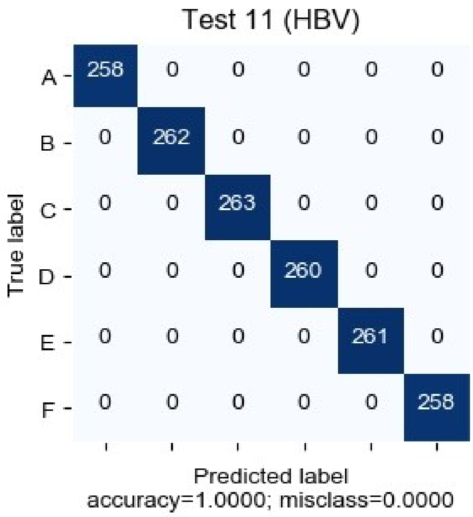
Test 11 (HBV)
True label
A 258 0 0 0 0 0
B 0 262 0 0 0 0
C 0 0 263 0 0 0
D 0 0 0 260 0 0
E 0 0 0 0 261 0
F 0 0 0 0 0 258
Predicted label
accuracy=1.0000; misclass=0.0000
